# Soft tissue tumor imaging in adults: whole-body staging in sarcoma, non-malignant entities requiring special algorithms, pitfalls and special imaging aspects. Guidelines 2024 from the European Society of Musculoskeletal Radiology (ESSR)

**DOI:** 10.1007/s00330-024-10897-z

**Published:** 2024-07-20

**Authors:** Iris-Melanie Noebauer-Huhmann, Filip M. Vanhoenacker, Joan C. Vilanova, Alberto S. Tagliafico, Marc-André Weber, Radhesh K. Lalam, Thomas Grieser, Violeta Vasilevska Nikodinovska, Jacky W. J. de Rooy, Olympia Papakonstantinou, Catherine Mccarthy, Luca Maria Sconfienza, Koenraad Verstraete, José Martel-Villagrán, Pavol Szomolanyi, Frédéric E. Lecouvet, Diana Afonso, Omar M. Albtoush, Giacomo Aringhieri, Remide Arkun, Gunnar Aström, Alberto Bazzocchi, Rajesh Botchu, Martin Breitenseher, Snehansh Chaudhary, Danoob Dalili, Mark Davies, Milko C. de Jonge, Berna D. Mete, Jan Fritz, Jan L. M. A. Gielen, Geoff Hide, Amanda Isaac, Slavcho Ivanoski, Ramy M. Mansour, Lorenzo Muntaner-Gimbernat, Ana Navas, Paul O´Donnell, Şebnem Örgüç, Winston J. Rennie, Santiago Resano, Philip Robinson, Hatice T. Sanal, Simone A. J. Ter Horst, Kirsten van Langevelde, Klaus Wörtler, Marita Koelz, Joannis Panotopoulos, Reinhard Windhager, Johan L. Bloem

**Affiliations:** 1 Department of Biomedical Imaging and Image Guided Therapy, Division of Neuroradiology and Musculoskeletal Radiology, Medical University of Vienna, Vienna, Austria; 2 Department of Radiology, AZ Sint Maarten Mechelen University (Hospital) Antwerp, Antwerp, Belgium; 3 Faculty of Medicine and Health Sciences, University of Ghent, Ghent, Belgium; 4 Department of Radiology, Clínica Girona, Institute of Diagnostic Imaging (IDI) Girona, University of Girona, Girona, Spain; 5 Department of Health Sciences (DISSAL), University of Genoa, Genoa, Italy; 6 Department of Radiology, IRCCS Ospedale Policlinico San Martino, Genoa, Italy; 7 Institute of Diagnostic and Interventional Radiology, Pediatric Radiology and Neuroradiology, University Medical Center Rostock, Rostock, Germany; 8 Department of Radiology, Robert Jones and Agnes Hunt Orthopaedic Hospital, Oswestry, UK; 9 Department for Diagnostic and Interventional Radiology, University Hospital Augsburg, Augsburg, Germany; 10 Medical Faculty, Ss. Cyril and Methodius University, Skopje, Macedonia; 11 Department of Radiology, University Surgical Clinic “St. Naum Ohridski”, Skopje, Macedonia; 12 Department of Imaging, Radiology, Radboud University Medical Center, Nijmegen, The Netherlands; 13 2nd Department of Radiology, Attikon Hospital, National and Kapodistrian University of Athens, Athens, Greece; 14 Oxford Musculoskeletal Radiology and Oxford University Hospitals, Oxford, UK; 15 IRCCS Istituto Ortopedico Galeazzi, Milan, Italy; 16 Dipartimento Di Scienze Biomediche Per La Salute, Università Degli Studi Di Milano, Milan, Italy; 17 Department of Radiology, Ghent University Hospital, Ghent, Belgium; 18 Radiology Department, Hospital Universitario Fundación Alcorcón, Madrid, Spain; 19 High Field MR Center, Department of Biomedical Imaging and Image‑Guided Therapy, Medical University Vienna, Vienna, Austria; 20 Department of Imaging Methods, Institute of Measurement Science, Slovak Academy of Sciences, Bratislava, Slovakia; 21 Department of Radiology and Medical Imaging, Cliniques Universitaires Saint Luc, Institut de Recherche Expérimentale et Clinique (IREC), Institut du Cancer Roi Albert II (IRA2), Université Catholique de Louvain (UCLouvain), Brussels, Belgium; 22 Hospital Particular da Madeira and Hospital da Luz Lisboa, Lisbon, Portugal; 23 Department of Radiology, University of Jordan, Ammam, Jordan; 24 Academic Radiology, Department of Translational Research and New Technologies in Medicine and Surgery, University of Pisa, Pisa, Italy; 25 Ege University Medical School (Emeritus), Izmir, Türkiye; 26 Star Imaging Center, Izmir, Türkiye; 27 Department of Immunology, Genetics and Pathology (Oncology) and Department of Surgical Sciences (Radiology), Uppsala University, Uppsala, Sweden; 28 Diagnostic and Interventional Radiology, IRCCS Istituto Ortopedico Rizzoli, Bologna, Italy; 29 Department of Musculoskeletal Radiology, Royal Orthopedic Hospital, Birmingham, UK; 30 Sigmund Freud Privatuniversität, Vienna, Austria; 31 Oxford University Hospitals NHS Foundation Trust, Oxford, UK; 32 Academic Surgical Unit, South West London Elective Orthopaedic Centre (SWLEOC), London, UK; 33 Department of Radiology, St. Antonius Hospital, Utrecht, The Netherlands; 34 Department of Radiology School of Medicine, Izmir Demokrasi University, Izmir, Türkiye; 35 Department of Radiology, NYU Grossman School of Medicine, New York, NY USA; 36 Diagnostic and Interventional Radiology, Eberhard Karls University Tuebingen, University Hospital Tuebingen, Tübingen, Germany; 37 Department of Radiology, Jessa Ziekenhuis, Campus Virga Jesse, Hasselt, Belgium; 38 Department of Radiology, Freeman Hospital, Newcastle Upon Tyne, UK; 39 School of Biomedical Engineering and Imaging Sciences, King’s College London, London, UK; 40 St. Erasmo Hospital for Orthopaedic Surgery and Traumatology Ohrid, Ohrid, Macedonia; 41 Oxford University Hospitals, Oxford, UK; 42 Hospital Univeritario Son Espases Balearic Islands University, Palma, Spain; 43 Department of Radiology, Leiden University Medical Center, Leiden, The Netherlands; 44 Royal National Orthopaedic Hospital, Stanmore, UK; 45 Manisa Celal Bayar University, Manisa, Türkiye; 46 Clinical MSK Radiology, Loughborough University, Leicester Royal Infirmary, Leicester, UK; 47 Hospital Universitario Ramón y Cajal, Madrid, Spain; 48 Musculoskeletal Radiology Department Chapel Allerton Hospital, Leeds Teaching Hospitals NHS Trust, Leeds, UK; 49 NIHR Leeds Biomedical Research Centre, Leeds, UK; 50 Radiology Department, University of Health Sciences, Gülhane Training and Research Hospital, Ankara, Türkiye; 51 Princess Máxima Center for Pediatric Oncology, Utrecht, The Netherlands; 52 Department of Radiology and Nuclear Medicine, University Medical Centre Utrecht, Utrecht, The Netherlands; 53 Musculoskeletal Radiology Section, Klinikum Rechts der Isar, Technical University of Munich ‑ TUM School of Medicine, Munich, Germany; 54 Clinical Institute of Pathology, Medical University of Vienna, Vienna, Austria; 55 Departement of Orthopaedics and Traumatology, Division of Orthopaedics, Medical University of Vienna, Vienna, Austria; 56 Departement of Orthopaedics and Traumatology, Medical University of Vienna, Vienna, Austria

**Keywords:** Practice guideline, Consensus, Neoplasms, Connective and soft tissue, Diagnostic imaging

## Abstract

**Objectives:**

The revised European Society of Musculoskeletal Radiology (ESSR) consensus guidelines on soft tissue tumor imaging represent an update of 2015 after technical advancements, further insights into specific entities, and revised World Health Organization (2020) and AJCC (2017) classifications. This second of three papers covers algorithms once histology is confirmed: (1) standardized whole-body staging, (2) special algorithms for non-malignant entities, and (3) multiplicity, genetic tumor syndromes, and pitfalls.

**Materials and methods:**

A validated Delphi method based on peer-reviewed literature was used to derive consensus among a panel of 46 specialized musculoskeletal radiologists from 12 European countries. Statements that had undergone interdisciplinary revision were scored online by the level of agreement (0 to 10) during two iterative rounds, that could result in ‘group consensus’, ‘group agreement’, or ‘lack of agreement’.

**Results:**

The three sections contain 24 statements with comments. Group consensus was reached in 95.8% and group agreement in 4.2%. For whole-body staging, pulmonary MDCT should be performed in all high-grade sarcomas. Whole-body MRI is preferred for staging bone metastasis, with [^18^F]FDG-PET/CT as an alternative modality in PET-avid tumors. Patients with alveolar soft part sarcoma, clear cell sarcoma, and angiosarcoma should be screened for brain metastases. Special algorithms are recommended for entities such as rhabdomyosarcoma, extraskeletal Ewing sarcoma, myxoid liposarcoma, and neurofibromatosis type 1 associated malignant peripheral nerve sheath tumors. Satisfaction of search should be avoided in potential multiplicity.

**Conclusion:**

Standardized whole-body staging includes pulmonary MDCT in all high-grade sarcomas; entity-dependent modifications and specific algorithms are recommended for sarcomas and non-malignant soft tissue tumors.

**Clinical relevance statement:**

These updated ESSR soft tissue tumor imaging guidelines aim to provide support in decision-making, helping to avoid common pitfalls, by providing general and entity-specific algorithms, techniques, and reporting recommendations for whole-body staging in sarcoma and non-malignant soft tissue tumors.

**Key Points:**

- *An early, accurate, diagnosis is crucial for the prognosis of patients with soft tissue tumors*.
- *These updated guidelines provide best practice expert consensus for standardized imaging algorithms, techniques, and reporting*.
- *Standardization can improve the comparability examinations and provide databases for large data analysis*.

## Introduction

Soft tissue tumors comprise a heterogeneous group of entities [1], which require histology-dependent standardized imaging algorithms. An early, accurate diagnosis is crucial, especially for the prognosis of these patients. At the same time, clinical infrastructure differs considerably throughout Europe. The same is true for the attitudes toward the use of advanced imaging techniques. This results in notable variability of soft tissue tumor imaging in clinical practice. Since the first consensus on soft tissue tumor imaging in adults of the European Society of Musculoskeletal Radiology (ESSR) in 2015 [2], technical advancements, further insights into specific entities, the revised World Health Organization classification (2020) [1], and a new version of the American Joint Committee on Cancer (AJCC) staging system (2017) [3] necessitated an update of the ESSR consensus guidelines [4]. The updated ESSR agreement for imaging of soft tissue tumors aims to provide best practice expert consensus guidelines for standardized imaging algorithms, techniques, and reporting in soft tissue tumors of adults. A Delphi process [5], evidence-based on current literature where possible, facilitates consensus on complex problems among a panel of experts [6] and has been used by several ESSR guidelines recently [7], including primary local imaging of soft tissue tumors [8].

This part of the recommendations is intended to support radiologists once the local staging has been completed and the histology has been confirmed. In patients with sarcoma, radiologists should be aware of current recommendation standards for whole-body staging in general, should know the entities in which a different approach has proved superior so far, and when additional imaging is necessary due to a different metastasis behavior. These consensus statements also provide guidance in some non-malignant entities. Other sections of this paper are dedicated to radiologic pitfalls that we have observed and how to avoid them. These include imaging of retroperitoneal liposarcomas and tumor-simulating masses. To prevent satisfaction of search (SOS), a list of syndromes that are associated with soft tissue tumors is also provided. We consider standardization once histology has been confirmed to be relevant both for better comparability of serial examinations in the individual patient, as well as for future large dataset evaluations in search of optimization of individualized patient care.

## Materials and methods

A validated Delphi method based on peer-reviewed literature, as has been described in detail in the first part of the ESSR consensus update on soft tissue tumor imaging [8], was used to derive consensus among a panel of 46 specialized musculoskeletal radiologists from 12 European countries, all being members of the tumor subcommittee of the ESSR. Institutional review board approval was not required for this consensus as patients were not involved. Statements were developed with comments, based on the current literature, by searching PubMed and the Cochrane Library. The statements were validated by two orthopedic tumor surgeons, a pathologist specializing in sarcoma, and a nuclear medicine expert. The panel members scored their level of agreement with each statement online by using an online questionnaire (Google Forms®) [9]. Suggestions for adjustments could be added and incorporated for the consecutive questionnaire round either as an alternative or an optimization of the statement. In three personal meetings, open questions and comments were discussed. The scores ranged from 0 to 10, with 10 being the highest grade of agreement. Minimum statement scoring required a median of at least eight and an interquartile range of less than four. For the statements which fulfilled these criteria, the level of agreement was calculated. “Group consensus” was defined as at least 80% of voters scoring at least eight and “Group agreement” was defined as 67–79% of voters scoring at least eight. “Lack of agreement” was assigned if the previous conditions were not met. After round 2 the rating was terminated for each statement.

## Results

This article contains three sections, with 24 statements overall. After round 2, group consensus was reached in 23/24 statements (95.8%), and group agreement was achieved in 1/24 statements (4.2%). None of the statements resulted in a lack of agreement.

The sections included (1) Whole-body staging in confirmed sarcoma, covering imaging algorithms and technical requirements (12 statements, all of them with group consensus, none with group agreement or with lack of agreement), (2) special algorithms for non-malignant entities (five statements, 5/0/0, respectively), (3) multiplicity, genetic tumor syndromes of soft tissue and pitfalls in soft tissue tumor imaging (seven statements, 6/1/0, respectively). Statements and their level of agreement are provided in Tables 1–3.

**Table 1:** Section 1. Whole-body staging in sarcoma. Statements

**1.1 Imaging methods for whole-body staging in sarcoma:**	**Median, IQR (difference (interval), Level of agreement**
**1.1.1 Generally appropriate imaging methods for whole-body staging in sarcoma:**	
‐ The most important radiological investigation for metastasis of soft-tissue sarcomas is unenhanced pulmonary MDCT. Pulmonary MDCT should be performed in all cases of high-grade sarcoma. Isotropic imaging with iterative reconstruction is favorable.	10; 0 (10–10); 100%
‐ MR imaging is the best method to depict skeletal metastases. Depending on the experience of the center, PET/CT can serve as an alternative in PET-avid tumors.	10; 2 (8–10); 91%
‐ Tumors likely to have lymphatic spread should be considered to be examined with contrast-enhanced MDCT of the abdomen and chest for lower extremity, and of the neck and chest for upper extremity sarcomas.	10; 2 (8–10); 84%
‐ FDG-PET/CT is helpful in individual sarcoma cases with lymph nodes in PET-avid tumors.	10; 1 (8–10); 93%
**1.1.2 Soft tissue sarcoma entities that require special imaging considerations for whole-body staging:**	
‐ Brain imaging should be performed using MRI in alveolar soft part sarcoma, clear cell sarcoma, and angiosarcoma. It may also be indicated in leiomyosarcoma, rhabdomyosarcoma, and spindle cell sarcoma.	10; 1 (9–10); 96%
‐ For the initial staging of younger rhabdomyosarcoma patients, whole-body FDG-PET/CT, or whole-body FDG-PET/MR imaging along with diagnostic chest CT are recommended.	10; 1 (9–10); 96%
‐ For the initial staging of patients with extraskeletal Ewing sarcoma, whole-body MRI along with diagnostic chest CT is recommended.	10; 2 (8–10); 97%
‐ Myxoid liposarcoma (MLS) has a propensity for extrapulmonary metastases. They are best staged by means of whole-body MR imaging (WB-MRI), which is therefore recommended.	10; 1 (9–10); 96%
‐ Whole-body MR imaging and whole-body FDG-PET/CT are useful in neurofibromatosis type 1 associated malignant peripheral nerve sheath tumors. Whole-body MR imaging is favored since the patients are not exposed to ionizing radiation.	10; 1 (9–10); 97%
**1.2 Imaging parameters for whole-body staging in sarcoma:**	
**1.2.1. PET/CT:**	
‐ FDG-PET-CT should be performed according to the latest version of the EANM protocol.	10; 1 (9–10); 91%
**1.2.2. Whole-body MRI:**	
‐ Whole-body MRI for soft tissue sarcomas should comprise a T1-weighted sequence and a fluid-sensitive T2-weighted fat-suppressed sequence as well as a diffusion-weighted sequence with the calculation of apparent diffusion coefficients.	10; 1 (9–10); 87%
‐ The diffusion-weighted sequence of the protocol should have at least two but optimally three *b*-values ranging from 50 to 900 s/mm^2^	10; 2 (8–10); 87%

**Table 2:** Section 2. Non-malignant entities that require special algorithms. Statements

**2.1. Nerve sheath tumors:**	**Median, IQR (difference (interval), Level of agreement**
‐ A watchful waiting approach for asymptomatic patients is recommended for neurofibromatosis (NF).	10; 1 (9–10); 93%
‐ Benign lesions that can often be diagnosed on US include peripheral nerve sheath tumors in case of proven neurofibromatosis for the detection and monitoring of typical neurofibromas. If painful, additional investigations should be conducted.	9; 2 (8–10); 81%
**2.2. Atypical lipomatous tumor (ALT) and well-differentiated liposarcoma (WDLS):**	
‐ Adipocytic tumors with the following features on MR (or CT) are suspicious for ALT/WDLS: Size > 11 cm, deep location, septa > 2 mm, septal enhancement, nodular areas. Location in the lower extremity also increases the likelihood for ALT/WDLS.	10; 1 (9–10); 91%
‐ For adipocytic superficial and extremity ALT, if not primarily resected, ultrasound follow-up is recommended.	10; 1 (9–10); 91%
‐ Adipocytic tumors that are located in the retroperitoneum or regions in which the tumor cannot be resected with a sufficient margin are termed WDLS. In case they are not resected, unenhanced MRI, or CT is preferred at yearly intervals or at the time when there are defined patient-reported outcome measures (PROM) such as the presence of increased pain, size, or tethering.	10; 1 (9–10); 91%

**Table 3:** Section 3. Pitfalls. Statements

**3.1. Soft tissue masses simulating tumors:**	**Median, IQR (difference (interval), Level of agreement**
‐ Soft tissue masses simulating tumors are common and should be kept in mind when evaluating US and MRI.	10; 1 (9–10); 95%
‐ Examples of such masses are anatomical variants, inflammatory, infectious, traumatic (nerve, muscle, reactive) skin lesions, metabolic lesions, and vascular lesions.	10; 1 (9–10); 95%
‐ CT scan can be a problem-solving modality in benign entities and tumor mimickers.	10; 0 (10–10); 100%
‐ Pitfalls include myositis ossificans or osseous entities that cause substantial soft tissue reactions, such as osteoid osteoma.	10; 0 (10–10); 100%
**3.2. Retroperitoneal liposarcoma:**	
‐ Pitfalls in local soft tissue tumor staging include the assessment of the extension of well-differentiated/dedifferentiated retroperitoneal liposarcoma: The well-differentiated part of the tumor appears equivalent or similar compared to normal fatty tissue of the retroperitoneum both in CT and MRI. Comparison with the contralateral side can be helpful.	10; 1 (9–10); 90%
**3.3. Consider potential multiplicity and syndromes:**	
‐ Beware of satisfaction of search (SOS): Vascular anomalies, lipoma, lipoma of tendon sheath, desmoid, neurofibroma, myxoma, and inclusion body fibromatosis may be multiple.	9; 2 (8–10); 76%
‐ Mafucci’s disease (hemangioma), Mazabraud (myxoma), Neurofibromatosis (schwannoma, neurofibroma), Gardner’s syndrome (fibromatosis), Turner’s syndrome (lymphangioma), Adenomatous polyposis (desmoid), Carney complex (myxoma) are syndromic associations with characteristic soft tissue lesions.	10; 2 (8–10); 87%

## Discussion

The updated ESSR consensus guidelines for soft tissue tumor imaging aim to provide feasible best practice expert state-of-the-art guidance. They are adjusted to the current literature, provide minimal requirements and an optimized strategy in a systematic approach, and contain relevant details. The Delphi process [10] was chosen as it allowed anonymous scoring [10]; a few additional face-to-face meetings proved useful for discussion of open questions regarding the procedure and of statements that had not reached consensus.

The expert panel was recruited from the dedicated musculoskeletal tumor subcommittee of the ESSR and included active representatives and soft tissue tumor imaging specialists from twelve European countries [11]. As group consensus was achieved in most statements, and group agreement in the remaining ones, this paper may help to provide feasible imaging algorithms taking into account different national infrastructures and approaches.

In the following paragraphs, we present a selection of the most clinically relevant statements with a short discussion (Table 1–3; additional comments are provided online as Supplementary Material).

### Whole-body staging in sarcoma

Section 1: (Table 1; for further comments please also see additional electronic material):

### General recommendations for whole-body staging in sarcoma

Metastatic spread of soft tissue sarcomas is mainly hematogenous, with a reported incidence of 11.9% in a surveillance, epidemiology, and end results (SEER) database based on data from 2000 to 2018 [12]. Overall, distant metastases are most common in the lungs, followed by bone, lymph nodes, liver, brain, and subcutaneous tissue [13]. With a 5–12-fold incidence, bone and lung metastases are more likely in sarcomas that are located underneath the deep fascia and in moderate or high-grade sarcomas [14]. The incidence of metastases is highly dependent on the histological tumor type [12, 13]. Metastases worsen the prognosis and result in upstaging in soft tissue sarcoma patients [15], while improved outcomes have been reported after metastasectomy [16]. Where appropriate, combinations of surgery, radiotherapy, and systemic treatment can significantly improve the prognosis of sarcoma [17]. Thus, diagnosis of metastases is important.

### Pulmonary metastases

Pulmonary metastatic disease at the time of diagnosis has been reported in 22% of patients with large (> 5 cm) high-grade soft tissue sarcomas of the extremities [18], and approximately 23% of patients with soft tissue sarcoma develop pulmonary metastases at some point of the disease course [19]. Computed tomography (CT) enables the detection of small pulmonary nodules [20], but is limited in its ability to differentiate between benign and malignant nodules [21]. In a retrospective study of high-grade sarcoma patients, CT revealed pulmonary nodules in 39.5% [21]. A total of 92% of the nodules > 5 mm were malignant, whereas 33% of nodules ≤ 5 mm and 20% ≤ 3 mm proved to be malignant [21]. In another study, the optimal threshold for a nodule at risk was 4.7 mm [22]. In this study utilizing FDG-PET/CT, the maximal standardized uptake value (SUV_max_) was significantly correlated with malignancy, with a specificity of 97.2%, but with a sensitivity of only 59.7%, FDG-PET/CT was considered unsatisfactory to differentiate metastatic from benign pulmonary nodules [22]. This was especially true for nodules < 5 mm, which were PET-positive in only 13.2% [22].

### Osseous metastases

The skeleton is the third most frequent site for metastases in soft tissue sarcomas, with reported rates of up to 10% [23]. In a SEER-based study on soft tissue sarcomas of the extremities, osseous metastases were found in 2.2% of patients at initial presentation [14]. Sarcoma grade [16, 23], location in the limb [23], especially the proximal limb [16], size > 5 cm [16], and regional lymph node involvement [14] were identified as risk factors for bone metastases. The spine is most affected [23]. The highest incidences have been described for alveolar soft part sarcomas [24, 25], angiosarcomas [23, 24], leiomyosarcomas [23, 26] (especially with combined osseous and lung metastases) [14], undifferentiated pleomorphic sarcomas [14, 23], myxoid liposarcoma [14, 27] and dedifferentiated liposarcomas [24]. Other entities which present with bone metastases were PNET (Ewing sarcoma), and synovial sarcoma [14]. Eighty percent of the osseous metastases are lytic [23, 28].

MR imaging showed higher sensitivity to detect bone metastases, compared to positron emission tomography (PET/CT) in a recent study on Ewing sarcoma patients, especially in widespread active hematopoietic bone marrow [29]. Due to the high soft tissue contrast of Magnetic resonance imaging (MRI), the use of contrast agents can often be avoided; MRI has proved especially useful for early detection of bone marrow involvement [30]. Another advantage of whole-body MRI is the lack of radiation exposure.

In a meta-analysis on bone metastases in different tumors, FDG-PET-CT had a sensitivity and specificity that was comparable to that of MRI, however superior to CT alone [31].

### Lymph node metastases

With about 4%, lymph node metastases are relatively uncommon in soft tissue sarcomas [32], except for a few subtypes. High prevalences have been observed in rhabdomyosarcoma (25.3–32.1%, even 54.8% in the alveolar type), clear cell sarcoma (15.9–27.7%), angiosarcoma (11.7–24.1%), and epithelioid sarcoma (12.4–31.8%) [12, 33–35]. In leiomyosarcoma (1.3–3.8%) and synovial sarcoma the prevalences are debated [12, 33, 34]. The presence of metastases to regional lymph nodes (N1) has also been associated with large and high-grade sarcomas and those located underneath the deep fascia [36], and nomograms have been developed to predict the likelihood of lymph node metastases [32].

Metastatic regional lymph nodes represent a strong prognostic factor [33]. In a study assessing extremity soft tissue sarcoma patients with isolated lymph node metastases, the prognosis for N1M0 was better than N0M1 [36], while it was similar in another study on soft tissue sarcomas [37]. The presence of lymph node metastases in the absence of M1 disease (N1M0), however, was associated with worse overall survival compared to N0M0 [35].

In the current 8th edition of the AJCC classification from 2017, in retroperitoneal sarcomas, N1M0 represents Grade IIIB, while in trunk and extremity soft tissue sarcoma N1 corresponds to Stage IV even in the absence of distant metastases [15].

The impact of PET/CT compared to conventional CT has not been finally clarified. In a multicentre study on pediatric sarcoma patients, FDG-PET revealed metastatic lymph nodes of rhabdomyosarcoma with a sensitivity of 93%, compared to 36% by conventional imaging modalities [38]. In the current National Comprehensive Cancer Network (NCCN) guidelines from 2023, CT or PET/CT is recommended for the assessment of regional lymph node basin in histologic tumor phenotypes at risk for lymph node metastases [15].

In general, PET/CT can serve as an alternative in PET-avid tumors treated with neoadjuvant therapy [15]. Of note, myxoid liposarcoma and synovial sarcoma metastases may have low FDG avidity which results in more false negative examinations compared to MR imaging [18].

### Soft tissue sarcoma entities that require special imaging considerations for whole-body staging

#### Brain imaging

Brain metastases in soft tissue sarcomas are rare at the time of diagnosis [39]. Their presence, however, worsens the prognosis considerably. Brain metastases occur more frequently in histologic soft tissue sarcoma subtypes such as alveolar soft part sarcoma (ASPS) [39–41], clear cell sarcoma, and angiosarcoma [42]. In those entities, brain imaging (MRI preferred over CT) should be performed [43]. Other subtypes with increased incidence include leiomyosarcoma and spindle cell sarcoma; occurrence in entities such as alveolar rhabdomyosarcoma and MPNST has been described [39]. Patients with high-grade or large tumors [44], and those with synchronous metastases, especially in the lung, bone, and lymph nodes are more likely to develop brain metastases [39, 45].

#### Myxoid liposarcoma (MLS)

Because of the unconventional metastatic behavior of Myxoid liposarcoma (MLS), with a high proportion of extrapulmonary metastases and low incidence of pulmonary metastases, and because of its low PET-avidity, whole-body MRI [46] is strongly recommended [27, 47, 48], both for early detection of bone and extraskeletal metastases [49] and for staging [50]. Comments to “Imaging parameters for whole-body staging in sarcoma” are provided online).

### Non-malignant entities that require special algorithms

Section 2: (Table 2; further comments are provided online):

#### Nerve sheath tumors

NF1 patients have approximately a 10% lifetime risk of acquiring this malignancy [51–53]. Peripheral nerve sheath tumors can be confirmed on Ultrasound (US) when the lesion is arising from a nerve, but clinical assessment is also vital: additional investigations should be conducted if the lesion is painful, growing rapidly, or in case of distal neurological dysfunction. Further imaging is usually also required in patients with NF1 [54]. In NF1, NF2, and schwannomatosis (SWN), emerging technical advances, particularly WB-MRI as well as DWI/ADC mapping, in conjunction with clinical and genetic data, can potentially provide insight into both disease severity as well as tumor behavior [55–57]. Similar accuracy in diagnosing malignant PNST has been reported for whole-body FDG-PET/CT and whole-body MR imaging [58]. PET/CT and MRI have complementary roles in MPNST evaluation: In several studies, PET was more sensitive while MRI offered higher specificity [59, 60]. WB PET/MR compared to PET/CT allowed the detection of PET-avid lesions with high accuracy, resulting in a reduction of radiation exposure of almost 50% [61], and therefore was considered a feasible alternative [61, 62].

### Pitfalls

Section 3: comments on the statements (listed in Table 3) are provided online.

### Limitations

As has been described earlier [8], this consensus has several limitations. The panelists came from European countries only. However, while access to modalities such as MRI and PET/CT is limited in many other parts of the world, this has to be taken into account only to a certain extent. In even less privileged countries, only some parts of this consensus will be currently applicable. Limitations of the Delphi method have been described earlier [8], including limited possibility for open discussion. On the other hand, all critical remarks could be considered anonymously without bias by dominant participants. The process was also time-consuming, which is a major disadvantage that has been described for guidelines that contain multiple statements, such as ours [10]. As high commitment was required for several questionnaire rounds, we aimed to provide sufficient time for the experts to answer. Finally, it should be emphasized that these guidelines reflect the current knowledge and will require further updates in the future. In particular, the field of radiomics and artificial intelligence is developing very rapidly.

## Conclusion

The updated ESSR guidelines for soft tissue tumor imaging regarding whole-body imaging in sarcoma, entity-dependent special algorithms for sarcomas and non-malignant soft tissue tumors in adults, and pitfalls provide best practice expert consensus for imaging and will support radiologists in their decision-making. Standardization may improve the comparability of serial examinations in the individual patient and may also provide databases for large data analysis aimed at developing individualized strategies.

## Supplementary information

Electronic Supplementary Material

## Abbreviations

AJCC: American Joint Committee on Cancer
CT: Computed tomography
ESSR: European Society of Musculoskeletal Radiology
MRI: Magnetic resonance imaging
PET/CT: Positron emission tomography
US: Ultrasound
