# Supplementary material for: Soft tissue tumor imaging in adults: whole-body staging in sarcoma, non-malignant entities requiring special algorithms, pitfalls and special imaging aspects. Guidelines 2024 from the European Society of Musculoskeletal Radiology (ESSR)
Source: Eur Radiol. 2024 Jul 20;35(1):351–9. doi: 10.1007/s00330-024-10897-z (PMC11631817; doi:10.1007/s00330-024-10897-z)
Supplement: Supplementary file 1 — Electronic Supplementary Material [file 330_2024_10897_MOESM1_ESM.pdf]

Soft tissue tumor imaging in adults: whole body staging in sarcoma, non-malignant entities requiring special algorithms, pitfalls and special imaging aspects. Guidelines 2024 from the European Society of Musculoskeletal Radiology (ESSR)

Electronic Supplementary Material (ESM)

Section 1. Whole body staging in sarcoma

1.1 Imaging methods for whole body staging in sarcoma:

1.1.1 Generally appropriate imaging methods:

- The most important radiological investigation for metastasis of soft-tissue sarcomas is unenhanced **pulmonary** MDCT. Pulmonary MDCT should be performed in all cases of high-grade sarcoma. Isotropic imaging with iterative reconstruction is favorable.
- MR imaging is the best method to depict **skeletal** metastases. Depending on the experience of the center, PET/CT can serve as an alternative in PET-avid tumors.
- Tumors likely to have **lymphatic** spread should be considered to be examined with contrast enhanced MDCT of the abdomen and chest for lower extremity, and of the neck and chest for upper extremity sarcomas.
- FDG PET/CT is helpful in individual sarcoma cases with lymph nodes in PET-avid tumors.

Comments:

Metastatic spread of soft tissue sarcomas is mainly hematogenous, with a reported incidence of 11.9% in a Surveillance, Epidemiology, and End Results (SEER) database based on data from 2000 to 2018 [1]. Overall, distant metastases are most common in the lungs, followed by bone, lymph nodes, liver, brain, and subcutaneous tissue [2]. With a 5-12-fold incidence, bone and lung metastases are more likely in sarcomas that are located underneath the deep fascia and in moderate or high-grade sarcomas [3]. The incidence of metastases is highly dependent on the histological tumor type [1, 2]. Metastases worsen the prognosis and lead to upstaging in soft tissue sarcoma patients [4]. Several studies report favourite outcomes by metastasectomy. For instance, in patients with metastatic extremity soft tissue sarcomas at the time of diagnosis, metastasectomy led to a better chance for prolonged survival [5].

Where appropriate, combinations of surgery, radiotherapy and systemic treatment can significantly improve the prognosis in sarcoma [6]. Thus, diagnosis of metastases is important.

Pulmonary metastases:

Pulmonary metastatic disease at the time of diagnosis has been reported in 22% of patients with large (>5cm) high grade soft tissue sarcomas of the extremities [7], and approximately 23% of patients with soft tissue sarcoma develop pulmonary metastases at some point of the disease course [8]. CT enables the detection of small pulmonary nodules [9], but is limited in its ability to differentiate between benign and malignant nodules [10]. In a retrospective study of high-grade sarcoma patients, CT revealed pulmonary nodules in 39.5% [10]. 92% of the nodules >5mm were malignant, whereas 33% of nodules ≤5mm and 20% ≤3mm proved to be malignant [10]. In another study, the optimal threshold for a nodule at risk was 4.7mm [11]. In this study utilizing FDG-PET/CT, the maximal standardized uptake value (SUVmax) was significantly correlated with malignancy, with a specificity of 97.2%. With a sensitivity of only 59.7%, FDG PET/CT was however considered unsatisfactory to

differentiate metastatic from benign pulmonary nodules [11]. This was especially true for nodules <5mm, which were PET positive in only 13.2% [11].

Osseous metastases:

The skeleton is the third most frequent site for metastases in soft tissue sarcomas, with reported rates of up to 10% [12]. In a SEER-based study on soft tissue sarcomas of the extremities, osseous metastases were found in 2.2% of patients at initial presentation [3]. They were more likely when regional lymph node involvement was present. With a 5-12-fold incidence, bone and lung metastases were more likely in deep and in moderate or high grade sarcomas [3]. Sarcoma grade [5, 12], location in the limb [12], especially the proximal limb [5] and size >5cm [5] were identified as risk factors for bone metastases in other studies. The spine is most affected [12].

The highest incidences have been described for alveolar soft part sarcomas [13, 14], angiosarcomas [12, 13], leiomyosarcomas [12, 15] (especially with combined osseous and lung metastases) [3], undifferentiated pleomorphic sarcomas [3, 12], myxoid liposarcoma [3, 16] and dedifferentiated liposarcomas [13]. Other entities which present with bone metastases were PNET (Ewing sarcoma), and synovial sarcoma [3]. Eighty percent of the osseous metastases are lytic [12, 17].

MR imaging showed higher sensitivity to detect bone metastases, compared to PET/CT in a recent study on Ewing sarcoma patients, especially in wide-spread active hematopoietic bone marrow [18]. Due to the high soft tissue contrast of MRI, the use of contrast agents can often be avoided; MRI has proved especially useful for early detection of bone marrow involvement [19]. Another advantage of whole-body MRI is the lack of radiation exposure.

In a meta-analysis on bone metastases in different tumors, FDG PET-CT had a sensitivity and specificity that was comparable to that of MRI, however superior to CT alone [20].

Lymph node metastases:

With about 4%, lymph node metastases are relatively uncommon in soft tissue sarcomas [21], except for a few subtypes. High prevalences have been observed in rhabdomyosarcoma (25.3%-32.1%, in alveolar rhabdomyosarcoma 54.8%), clear cell sarcoma (15.9%-27.7%), angiosarcoma (11.7-24.1%), epithelioid sarcoma (12.4%-31.8%) [1, 22-24]. In leiomyosarcoma (1.3%-3.8%) and synovial sarcoma the prevalences are debated [1, 22, 23]. The presence of metastases to regional lymph nodes (N1) has also been associated with large and high-grade sarcomas and those located underneath the deep fascia [25], and nomograms have been developed to predict the likelihood of lymph node metastases [21].

Metastatic regional lymph nodes represent a strong prognostic factor [22]. In a study assessing extremity soft tissue sarcoma patients with isolated lymph node metastases, the prognosis for N1M0 was better than N0M1 [25], while it was similar in another study on soft tissue sarcomas [26]. The presence of lymph node metastases in the absence of M1 disease (N1M0), however, was associated with worse overall survival compared to N0M0 [24].

In the current 8th ed. of the AJCC-classification from 2017, in retroperitoneal sarcomas N1 M0 represents Grade IIIB, while in trunk and extremity soft tissue sarcoma N1 corresponds to Stage IV even in the absence of distant metastases [4, 27].

Due to the prognostic impact, CT staging should therefore include the regional lymph node levels (at least if they are not covered by the regional MRI).
The impact of PET/CT compared to conventional CT has not been finally clarified. In a multicentre study on pediatric sarcoma patients, FDG-PET revealed metastatic lymph nodes of rhabdomyosarcoma with a sensitivity of 93%, compared to 36% by conventional imaging modalities [28].
It has to be kept in mind that sarcoma patients (as all patients) may simultaneously suffer from other diseases that affect lymph nodes and can cause elevated SUV with false positive PET/CT results (including infections and other inflammatory changes, such as sarcoidosis in mediastinal lymph nodes) [29]. On the other hand, small lymph node size (< 5mm) may lead to false negative results; hyperglycemia during the examination has to be avoided (please see section on technique).
In the current National Comprehensive Cancer Network (NCCN) guidelines from 2023, CT or PET/CT is recommended for the assessment of regional lymph node basin in histologic tumor phenotypes at risk for lymph node metastases [4].

In general, PET/CT can serve as an alternative modality in PET-avid tumors, and is considered potentially useful for staging in histologies in which neoadjuvant therapy is being used [4].
Of note, myxoid liposarcoma and synovial sarcoma metastases may have low FDG avidity which results in more false negative examinations compared to MR imaging [7].

1.1.2 Soft tissue sarcoma entities that require special imaging considerations for whole body staging.

- **Brain imaging** should be performed using MRI in alveolar soft part sarcoma, clear cell sarcoma, and angiosarcoma. It may also be indicated in leiomyosarcoma, rhabdomyosarcoma, and spindle cell sarcoma.
- For the initial staging of younger **rhabdomyosarcoma** patients, whole body FDG-PET/CT, or whole-body FDG-PET/MR imaging along with diagnostic chest CT are recommended.
- For the initial staging of patients with **extraskeletal Ewing sarcoma**, whole body MRI along with diagnostic chest CT is recommended.
- **Myxoid liposarcoma** (MLS) has a propensity for extra-pulmonary metastases. They are best staged by means of whole-body MR imaging (WB-MRI), which is therefore recommended.

Comments:

Brain imaging

Brain metastases in soft tissue sarcomas are rare at the time of diagnosis [30]. Their presence, however, worsens the prognosis considerably. Brain metastases occur more frequently in histologic

soft tissue sarcoma subtypes such as alveolar soft part sarcoma (ASPS) [30-32], clear cell sarcoma, and angiosarcoma [33]; in those entities, brain imaging should be performed [34]. Other subtypes with increased incidence include leiomyosarcoma and spindle cell sarcoma; occurrence in entities such as alveolar rhabdomyosarcoma and MPNST has been described [30]. Patients with high grade or large tumors [35], and those with synchronous metastases, especially in the lung, bone and lymph nodes are more likely to develop brain metastases [30, 36].

Rhabdomyosarcoma (RMS)

Rhabdomyosarcoma is rare in adults, and histological subtypes are common that are unusual during childhood, such as the not otherwise specified (NOS) (13%- 43.3%) [37, 38] and the pleomorphic subtype (19.1%-36%)[37-39]. The prevalence of the embryonal and the alveolar subtype in adults are 17%-32% [37, 38, 40] and 18%-35% [37, 38], respectively. The prognosis of adult rhabdomyosarcoma is significantly worse than that of pediatric patients [38], with a 5-year overall-survival (OAS) rate of about 27%- 30% (compared to 61% in children) [37, 41].

Lymph node involvement and metastases are frequently encountered: In a large SEER-based study, regional spread (infiltration of adjacent tissue or involvement of regional lymph nodes) was revealed in 25.5% [37]. In another study, even 37% of the patients had lymph node involvement at the time of diagnosis [40].

Distant metastases were reported in 17- 28.2% of the patients [37, 40]. The most common sites of metastatic spread are the lungs and the bone marrow.

The SEER-based stage is a significant predictor of survival [37]. The hazard ratios for death in case of regional spread and distant metastases vs. localized disease were 1.7 and 4.3, respectively [37]. Reported five-year OAS was 40% for localized disease versus 15% for metastatic disease [41]. Whole-body staging is therefore essential in adult rhabdomyosarcoma.

In children, ^{18}F- PET/CT has shown a higher sensitivity in the detection of involved lymph nodes and skeletal metastases, compared to conventional imaging (CI; consisting of MRI and/or CT of the primary tumor, chest CT and bone scintigraphy)[42]. The sensitivity to detect pulmonary metastases was lower in that study, stressing the importance of diagnostic quality of the CT component in PET/CT [42]. In another study on both children and adults, the accuracy ^{18}F- PET/CT in staging rhabdomyosarcoma was also superior to CI, with 97% vs. 87% [43]. Moreover, local SUV_{max} seems to be an additional prognostic parameter, and a decrease under therapy could early identify patients who would respond to systemic treatment [44]. To date, the availability of PET/MR is limited. However PET/MR results in significantly less radiation exposure compared to PET/CT [45].

Recently, for children and adolescent patients with rhabdomyosarcoma, chest CT along with ^{18}F-PET/CT or PET/MRI were recommended for the detection and evaluation of loco-regional and distant metastases [46].

Extraskeletal Ewing sarcoma (EES)

In contrast to the pediatric manifestation, over half of adult Ewing sarcomas are extraskeletal [47]. With a median age of 20 years, patients are slightly older than those affected by osseous Ewing, and EES arise in axial locations more often (about 73%) [48]. EES are high grade sarcomas, and rates of

gross metastases between 15% and 46% have been reported [49]. Micrometastatic disease in virtually all patients at the time of diagnosis has been postulated [50]; this assumption is supported by a very low cure rate in EES patients who only had received local treatment [50].

With up to 27%, the lung is the most common site for metastases of EES [51].

Of note, compared to Ewing sarcoma of the bone (ESB), lymph node involvement is more frequent in EES [51] and has been described in up to more than 75% [52].

Bone and bone marrow metastases are also seen [51-53]. Metastases to the liver, brain, peritoneum and pleura are seen less frequently [51-53]. Soft tissue metastases and metastases in the peritoneal cavity have also been reported [51].

As a consequence, FDG-PET/CT is increasingly used for whole body staging in adult patients with EES [54, 55]. A meta-analysis on Ewing sarcoma family of tumors (ESFT) that reviewed 23 studies and included extraskeletal Ewing sarcomas [56] found high diagnostic odds ratios (DOR) of 18F-FDG PET and PET/CT for lung and bone metastases. For the assessment of pulmonary metastases, the chest CT has to be of diagnostic quality [57].

Whole body MRI (WB-MRI) bears the advantage that radiation exposure can be avoided; and the tissue contrast is excellent. It is less commonly performed, which may also be due to a limited availability and long examination time [55], and the necessity to perform an additional chest CT. In a systematic review, with 82-100%, WB-MRI showed high sensitivity for the detection of skeletal metastases in solid tumors (40,6% of them with ES) [58], WB-MRI and/or FDG-PET/CT are considered comparable for the detection of bone marrow metastases in children and young adults [59].

Reported sensitivity, specificity, and accuracy in the general assessment of lesions in Ewing sarcoma patients by FDG-PET/CT were 87%, 97%, and 94% [57].

The PET aviditiy can also be used as a quantitative parameter for disease activity and, later, treatment response [51].

Myxoid liposarcoma (MLS):

Because of the unconventional metastatic behavior of MLS, with a high proportion of extrapulmonary metastases and low incidence of pulmonary metastases, and because of its low PET-avidity, whole-body MRI is strongly recommended [16, 60, 61]. It must be kept in mind that CT and PET-CT are both of inferior value to detect metastastic osseous involvement due to lack of bony destructions and reduced FDGavidity [62]. Osseous metastases predominate, with metastases in the spine, the pelvis, the chest-wall and long bones being the most frequent ones, followed by soft-tissue and abdominal lesions; lymph node involvement may be present [61, 63, 64]. WBMRI has been recommended both for early detection of bone and extra-skeletal metastases [65] and for staging [63, 66, 67]. A possible MRI protocol contains at least coronal and axial STIR and a coronal T1w sequence [66]. A potential protocol for whole body staging could include whole body MRI and chest CT although it is still unclear whether WB-MRI can replace contrast-enhanced CT of the abdominopelvic region [62].

Role of Whole-body MRI and PET/MRI:

Whole-body magnetic resonance imaging (WB-MRI) is gradually being integrated into clinical pathways for the detection, characterization, and staging of malignant tumours including those arising

in the musculoskeletal (MSK) system. Although further developments and research are needed, it is now recognized that WB-MRI enables reliable, sensitive, and specific detection and quantification of disease burden for a variety of disease types and a particular application for skeletal involvement. Advances in imaging techniques now allow the reliable incorporation of WB-MRI into clinical pathways, and guidelines recommending its use are emerging [68].
In patients with PET positive sarcomas, the use of whole-body PET/MR is promising [69], and also carries the advantage of significantly less radiation exposure compared to PET/CT [45]. To date, due to limited availability and high costs, it is not utilized widely. However, guidelines providing detailed recommendations on all important technical aspects have been provided recently, and allow standardized examinations [69]. With further technical advance, its inclusion in whole body imaging algorithms in special sarcoma entities is expected in future.

1.2 Imaging parameters for whole body staging in sarcoma:

1.2.1. PET/CT:

- FDG-PET-CT should be performed according to the latest version of the EANM protocol.

1.2.2. Whole-body MRI:

- Whole-body MRI for soft tissue sarcomas should comprise a T1-weighted sequence and a fluid-sensitive T2-weighted fat suppressed sequence as well as a diffusion weighted sequence with the calculation of apparent diffusion coefficients.
- The diffusion-weighted sequence of the protocol should have at least two but optimally three b-values ranging from 50 to 900 s/mm2.

The European Association of Nuclear Medicine (EANM) provides recommendations for all technical aspects on PET/CT with regular updates [70]. Whole body imaging in soft tissue sarcoma patients should be performed according tho the guidelines of this society [70].
WB-MRI with apparent diffusion coefficient (ADC) mapping enhances disease detection and response assessment performance of WB-MRI. It combines anatomical localization, morphological evaluation, tumor size and volume of standard anatomical sequences with functional evaluations of the underlying tumour matrix including the depiction of cellularity and necrosis, as well as quantification of bone marrow fat, while providing high image contrast between tumours and healthy tissues, alleviating the need for administration of gadoliniumbased contrast medium in most cases [68]. However, there is no consensus on the cut-off value of ADC value [71].
The technical parameters of the diffusion weighted images should be: axial diffusion weighted sequences (typical b-values of 50, 400–600, and 900 s/mm^2) [68], with corresponding ADC maps covering vertex to feet if clinically indicated (e.g., known metastasis beyond coverage 1, primary sarcoma, neurofibromatosis-1 [72, 73], Li-Fraumeni syndrome) [74-76]. The rationale for implementing this technique is: 1) To depict and quantify abnormal cellularity within bone marrow, 2) To facilitate

assessment of disease response through quantification of disease cellularity, 3) To assess associated soft tissue disease presence, extent, and its response to therapy.

REFERENCES

1. *Liu H, Zhang H, Zhang C, et al. (2022) Pan-Soft Tissue Sarcoma Analysis of the Incidence, Survival, and Metastasis: A Population-Based Study Focusing on Distant Metastasis and Lymph Node Metastasis. Front Oncol 12:890040*
2. *Trovik C, Bauer HCF, Styring E, et al. (2017) The Scandinavian Sarcoma Group Central Register: 6,000 patients after 25 years of monitoring of referral and treatment of extremity and trunk wall soft-tissue sarcoma. Acta Orthop 88(3):341-347*
3. *Younis MH, Summers S and Pretell-Mazzini J (2022) Bone metastasis in extremity soft tissue sarcomas: risk factors and survival analysis using the SEER registry. Musculoskelet Surg 106(1):59-68*
4. *Sarcoma NCPGiOST (2023) Network Version 2.2023. J Natl Compr Canc Netw*
5. *Krishnan CK, Kim HS, Park JW and Han I (2018) Outcome After Surgery for Extremity Soft Tissue Sarcoma in Patients Presenting With Metastasis at Diagnosis. Am J Clin Oncol 41(7):681-686*
6. *Gonzalez MR, Inchaustegui ML, Ruiz-Arellanos K, de Souza FF, Subhawong TK and Pretell-Mazzini J (2023) Management of oligometastatic disease in soft tissue sarcomas. Journal of Cancer Metastasis and Treatment 9:12*
7. *Sambri A, Bianchi G, Longhi A, et al. (2019) The role of 18F-FDG PET/CT in soft tissue sarcoma. Nucl Med Commun 40(6):626-631*
8. *Billingsley KG, Burt ME, Jara E, et al. (1999) Pulmonary metastases from soft tissue sarcoma: analysis of patterns of diseases and postmetastasis survival. Ann Surg 229(5):602-10; discussion 610-2*
9. *Hanamiya M, Aoki T, Yamashita Y, Kawanami S and Korogi Y (2012) Frequency and significance of pulmonary nodules on thin-section CT in patients with extrapulmonary malignant neoplasms. Eur J Radiol 81(1):152-7*
10. *Nakamura T, Matsumine A, Matsusaka M, et al. (2017) Analysis of pulmonary nodules in patients with high-grade soft tissue sarcomas. PLoS One 12(2):e0172148*
11. *Hagi T, Nakamura T, Sugino Y, Matsubara T, Asanuma K and Sudo A (2018) Is FDG-PET/CT Useful for Diagnosing Pulmonary Metastasis in Patients with Soft Tissue Sarcoma? Anticancer Res 38(6):3635-3639*
12. *Vincenzi B, Frezza AM, Schiavon G, et al. (2013) Bone metastases in soft tissue sarcoma: a survey of natural history, prognostic value and treatment options. Clin Sarcoma Res 3(1):6*
13. *Yoshikawa H, Myoui A, Ochi T, et al. (1999) Bone Metastases from Soft Tissue Sarcomas. Semin Musculoskelet Radiol 3(2):183-190*
14. *Sood S, Baheti AD, Shinagare AB, et al. (2014) Imaging features of primary and metastatic alveolar soft part sarcoma: single institute experience in 25 patients. Br J Radiol 87(1036):20130719*
15. *Gordon RW, Tirumani SH, Kurra V, et al. (2014) MRI, MDCT features, and clinical outcome of extremity leiomyosarcomas: experience in 47 patients. Skeletal Radiol 43(5):615-22*
16. *Schwab JH, Boland PJ, Antonescu C, Bilsky MH and Healey JH (2007) Spinal metastases from myxoid liposarcoma warrant screening with magnetic resonance imaging. Cancer 110(8):1815-22*
17. *Wong WS, Kaiser LR, Gold RH and Fon GT (1982) Radiographic features of osseous metastases of soft-tissue sarcomas. Radiology 143(1):71-4*
18. *Bosma SE, Vriens D, Gelderblom H, van de Sande MAJ, Dijkstra PDS and Bloem JL (2019) (18)F-FDG PET-CT versus MRI for detection of skeletal metastasis in Ewing sarcoma. Skeletal Radiol 48(11):1735-1746*
19. *O'Sullivan GJ, Carty FL and Cronin CG (2015) Imaging of bone metastasis: An update. World J Radiol 7(8):202-11*

20. *Yang HL, Liu T, Wang XM, Xu Y and Deng SM (2011) Diagnosis of bone metastases: a meta-analysis comparing (1)(8)FDG PET, CT, MRI and bone scintigraphy. Eur Radiol 21(12):2604-17*
21. *Tong Y, Pi Y, Cui Y, Jiang L, Gong Y and Zhao D (2022) Early distinction of lymph node metastasis in patients with soft tissue sarcoma and individualized survival prediction using the online available nomograms: A population-based analysis. Front Oncol 12:959804*
22. *Jacobs AJ, Morris CD and Levin AS (2018) Synovial Sarcoma Is Not Associated With a Higher Risk of Lymph Node Metastasis Compared With Other Soft Tissue Sarcomas. Clin Orthop Relat Res 476(3):589-598*
23. *Sherman KL, Kinnier CV, Farina DA, et al. (2014) Examination of national lymph node evaluation practices for adult extremity soft tissue sarcoma. J Surg Oncol 110(6):682-8*
24. *Keung EZ, Chiang YJ, Voss RK, et al. (2018) Defining the incidence and clinical significance of lymph node metastasis in soft tissue sarcoma. Eur J Surg Oncol 44(1):170-177*
25. *Basile G, Mattei JC, Alshaygy I, et al. (2020) Curability of patients with lymph node metastases from extremity soft-tissue sarcoma. Cancer 126(23):5098-5108*
26. *Garcia-Ortega DY, Alvarez-Cano A, Clara-Altamirano MA, et al. (2021) Should metastatic lymph nodes be considered at the same clinical stage as distant metastasis in soft tissue sarcomas? Cancer Treat Res Commun 26:100268*
27. *Amin MB, Greene FL, Edge SB, et al. (2017) The Eighth Edition AJCC Cancer Staging Manual: Continuing to build a bridge from a population-based to a more "personalized" approach to cancer staging. CA Cancer J Clin 67(2):93-99*
28. *Volker T, Denecke T, Steffen I, et al. (2007) Positron emission tomography for staging of pediatric sarcoma patients: results of a prospective multicenter trial. J Clin Oncol 25(34):5435-41*
29. *Rayamajhi SJ, Mittal BR, Maturu VN, et al. (2016) (18)F-FDG and (18)F-FLT PET/CT imaging in the characterization of mediastinal lymph nodes. Ann Nucl Med 30(3):207-16*
30. *Gonzalez MR, Bryce-Alberti M, Leon-Abarca JA and Pretell-Mazzini J (2021) Brain Metastases in Patients with Soft-Tissue Sarcomas: Management and Survival-A SEER Population-Based Cohort Study. Journal of the American Academy of Orthopaedic Surgeons Global Research and Reviews 5(10)*
31. *Lieberman PH, Brennan MF, Kimmel M, Erlandson RA, Garin-Chesa P and Flehinger BY (1989) Alveolar soft-part sarcoma. A clinico-pathologic study of half a century. Cancer 63(1):1-13*
32. *Portera CA, Jr., Ho V, Patel SR, et al. (2001) Alveolar soft part sarcoma: clinical course and patterns of metastasis in 70 patients treated at a single institution. Cancer 91(3):585-91*
33. *Meis-Kindblom JM and Kindblom LG (1998) Angiosarcoma of soft tissue: a study of 80 cases. Am J Surg Pathol 22(6):683-97*
34. *Espat NJ, Bilsky M, Lewis JJ, Leung D and Brennan MF (2002) Soft tissue sarcoma brain metastases. Prevalence in a cohort of 3829 patients. Cancer 94(10):2706-11*
35. *Chaigneau L, Patrikidou A, Ray-Coquard I, et al. (2018) Brain Metastases from Adult Sarcoma: Prognostic Factors and Impact of Treatment. A Retrospective Analysis from the French Sarcoma Group (GSF/GETO). Oncologist 23(8):948-955*
36. *Chan CM, Lindsay AD, Spiguel AR, Scarborough MT and Gibbs CP (2020) Brain metastases from Truncal and extremity bone and soft tissue sarcoma: Single institution study of oncologic outcomes. Rare Tumors 12:2036361320960060*
37. *Sultan I, Qaddoumi I, Yaser S, Rodriguez-Galindo C and Ferrari A (2009) Comparing adult and pediatric rhabdomyosarcoma in the surveillance, epidemiology and end results program, 1973 to 2005: an analysis of 2,600 patients. J Clin Oncol 27(20):3391-7*
38. *Bompas E, Campion L, Italiano A, et al. (2018) Outcome of 449 adult patients with rhabdomyosarcoma: an observational ambispective nationwide study. Cancer Med 7(8):4023-4035*
39. *Saadi M, Letaief F, Gabsi A, Mokrani A, Meddeb K and Mezlini A (2022) Clinical and pathological characteristics, treatment outcome and prognostic factors in adult rhabdomyosarcoma: a monocentric retrospective study. Pan Afr Med J 41:246*

40. *Ferrari A, Dileo P, Casanova M, et al. (2003) Rhabdomyosarcoma in adults. A retrospective analysis of 171 patients treated at a single institution. Cancer 98(3):571-80*
41. *Makinen VN, Safwat A and Aggerholm-Pedersen N (2021) Rhabdomyosarcoma in Adults: A Retrospective Analysis of Case Records Diagnosed between 1979 and 2018 in Western Denmark. Sarcoma 2021:9948885*
42. *Mercolini F, Zucchetta P, Jehanno N, et al. (2021) Role of (18)F-FDG-PET/CT in the staging of metastatic rhabdomyosarcoma: a report from the European paediatric Soft tissue sarcoma Study Group. Eur J Cancer 155:155-162*
43. *Tateishi U, Hosono A, Makimoto A, et al. (2009) Comparative study of FDG PET/CT and conventional imaging in the staging of rhabdomyosarcoma. Ann Nucl Med 23(2):155-61*
44. *Lim HJ, Johnny Ong CA, Tan JW and Ching Teo MC (2019) Utility of positron emission tomography/computed tomography (PET/CT) imaging in the evaluation of sarcomas: A systematic review. Crit Rev Oncol Hematol 143:1-13*
45. *Gennaro N, Marrari A, Renne SL, et al. (2020) Multimodality imaging of adult rhabdomyosarcoma: the added value of hybrid imaging. Br J Radiol 93(1112):20200250*
46. *van Ewijk R, Schoot RA, Sparber-Sauer M, et al. (2021) European guideline for imaging in paediatric and adolescent rhabdomyosarcoma - joint statement by the European Paediatric Soft Tissue Sarcoma Study Group, the Cooperative Weichteilsarkom Studiengruppe and the Oncology Task Force of the European Society of Paediatric Radiology. Pediatr Radiol 51(10):1940-1951*
47. *Maki RG (2008) Pediatric sarcomas occurring in adults. J Surg Oncol 97(4):360-8*
48. *Applebaum MA, Worch J, Matthay KK, et al. (2011) Clinical features and outcomes in patients with extraskeletal Ewing sarcoma. Cancer 117(13):3027-32*
49. *Javery O, Krajewski K, O'Regan K, et al. (2011) A to Z of extraskeletal Ewing sarcoma family of tumors in adults: imaging features of primary disease, metastatic patterns, and treatment responses. AJR Am J Roentgenol 197(6):W1015-22*
50. *Ludwig JA (2008) Ewing sarcoma: historical perspectives, current state-of-the-art, and opportunities for targeted therapy in the future. Curr Opin Oncol 20(4):412-8*
51. *Somarouthu BS, Shinagare AB, Rosenthal MH, et al. (2014) Multimodality imaging features, metastatic pattern and clinical outcome in adult extraskeletal Ewing sarcoma: experience in 26 patients. Br J Radiol 87(1038):20140123*
52. *Huh J, Kim KW, Park SJ, et al. (2015) Imaging Features of Primary Tumors and Metastatic Patterns of the Extraskeletal Ewing Sarcoma Family of Tumors in Adults: A 17-Year Experience at a Single Institution. Korean J Radiol 16(4):783-90*
53. *Haeusler J, Ranft A, Boelling T, et al. (2010) The value of local treatment in patients with primary, disseminated, multifocal Ewing sarcoma (PDMES). Cancer 116(2):443-50*
54. *Cesari M, Righi A, Colangeli M, et al. (2019) Bone marrow biopsy in the initial staging of Ewing sarcoma: Experience from a single institution. Pediatr Blood Cancer 66(6):e27653*
55. *Wright A, Desai M, Bolan CW, et al. (2022) Extraskeletal Ewing Sarcoma from Head to Toe: Multimodality Imaging Review. Radiographics 42(4):1145-1160*
56. *Huang T, Li F, Yan Z, et al. (2018) Effectiveness of 18F-FDG PET/CT in the diagnosis, staging and recurrence monitoring of Ewing sarcoma family of tumors: A meta-analysis of 23 studies. Medicine (Baltimore) 97(48):e13457*
57. *Gerth HU, Juergens KU, Dirksen U, Gerss J, Schober O and Franzius C (2007) Significant benefit of multimodal imaging: PET/CT compared with PET alone in staging and follow-up of patients with Ewing tumors. J Nucl Med 48(12):1932-9*
58. *Smets AM, Deurloo EE, Slager TJE, Stoker J and Bipat S (2018) Whole-body magnetic resonance imaging for detection of skeletal metastases in children and young people with primary solid tumors - systematic review. Pediatr Radiol 48(2):241-252*
59. *Ingley KM, Wan S, Voo S, et al. (2021) Is It Time to Call Time on Bone Marrow Biopsy for Staging Ewing Sarcoma (ES)? Cancers (Basel) 13(13)*

60. *Lin S, Gan Z, Han K, Yao Y and Min D (2015) Metastasis of myxoid liposarcoma to fat-bearing areas: A case report of unusual metastatic sites and a hypothesis. Oncol Lett 10(4):2543-2546*
61. *Durr HR, Rauh J, Baur-Melnyk A, et al. (2018) Myxoid liposarcoma: local relapse and metastatic pattern in 43 patients. BMC Cancer 18(1):304*
62. *Saifuddin A, Andrei V, Rajakulasingam R, Oliveira I and Seddon B (2021) Magnetic resonance imaging of trunk and extremity myxoid liposarcoma: diagnosis, staging, and response to treatment. Skeletal Radiol 50(10):1963-1980*
63. *Stevenson JD, Watson JJ, Cool P, et al. (2016) Whole-body magnetic resonance imaging in myxoid liposarcoma: A useful adjunct for the detection of extra-pulmonary metastatic disease. Eur J Surg Oncol 42(4):574-80*
64. *Mujtaba B, Wang F, Taher A, Aslam R, Madewell JE and Nassar S (2021) Myxoid Liposarcoma With Skeletal Metastases: Pathophysiology and Imaging Characteristics. Curr Probl Diagn Radiol 50(1):66-73*
65. *Gouin F, Renault A, Bertrand-Vasseur A, et al. (2019) Early detection of multiple bone and extra-skeletal metastases by body magnetic resonance imaging (BMRI) after treatment of Myxoid/Round-Cell Liposarcoma (MRCLS). Eur J Surg Oncol 45(12):2431-2436*
66. *Gorelik N, Reddy SMV, Turcotte RE, et al. (2018) Early detection of metastases using whole-body MRI for initial staging and routine follow-up of myxoid liposarcoma. Skeletal Radiol 47(3):369-379*
67. *Seo SW, Kwon JW, Jang SW, Jang SP and Park YS (2011) Feasibility of whole-body MRI for detecting metastatic myxoid liposarcoma: a case series. Orthopedics 34(11):e748-54*
68. *Isaac A, Lecouvet F, Dalili D, et al. (2020) Detection and Characterization of Musculoskeletal Cancer Using Whole-Body Magnetic Resonance Imaging. Semin Musculoskelet Radiol 24(6):726-750*
69. *Umutlu L, Beyer T, Grueneisen JS, et al. (2019) Whole-Body [18F]-FDG-PET/MRI for Oncology: A Consensus Recommendation. Rofo 191(4):289-297*
70. *Boellaard R, Delgado-Bolton R, Oyen WJG, et al. (2015) FDG PET/CT: EANM procedure guidelines for tumour imaging: version 2.0. European Journal of Nuclear Medicine and Molecular Imaging 42(2):328-354*
71. *Ahlawat S, Fritz J, Morris CD and Fayad LM (2019) Magnetic resonance imaging biomarkers in musculoskeletal soft tissue tumors: Review of conventional features and focus on nonmorphologic imaging. J Magn Reson Imaging 50(1):11-27*
72. *Vilanova JC, Garcia-Figueiras R, Luna A, Baleato-Gonzalez S, Tomas X and Narvaez JA (2019) Update on Whole-body MRI in Musculoskeletal Applications. Semin Musculoskelet Radiol 23(3):312-323*
73. *Ahlawat S, Blakeley JO, Langmead S, Belzberg AJ and Fayad LM (2020) Current status and recommendations for imaging in neurofibromatosis type 1, neurofibromatosis type 2, and schwannomatosis. Skeletal Radiol 49(2):199-219*
74. *Consul N, Amini B, Ibarra-Rovira JJ, et al. (2021) Li-Fraumeni Syndrome and Whole-Body MRI Screening: Screening Guidelines, Imaging Features, and Impact on Patient Management. AJR Am J Roentgenol 216(1):252-263*
75. *O'Neill AF, Voss SD, Jagannathan JP, et al. (2018) Screening with whole-body magnetic resonance imaging in pediatric subjects with Li-Fraumeni syndrome: A single institution pilot study. Pediatr Blood Cancer 65(2)*
76. *Bojadzieva J, Amini B, Day SF, et al. (2018) Whole body magnetic resonance imaging (WB-MRI) and brain MRI baseline surveillance in TP53 germline mutation carriers: experience from the Li-Fraumeni Syndrome Education and Early Detection (LEAD) clinic. Fam Cancer 17(2):287-294*

Section 2. Non-malignant entities that require special algorithms

2.1. Nerve sheath tumors

- A watchful waiting approach for asymptomatic patients is recommended for neurofibromatosis (NF).
- Benign lesions that can often be diagnosed on US include peripheral nerve sheath tumours in case of proven neurofibromatosis for the detection and monitoring of typical neurofibromas. If painful, additional investigations should be conducted.

Approximately half of malignant peripheral nerve sheath tumours (MPNST) occur in the context of NF1 which is one of the most important prognostic factors. NF1 patients have approximately a 10% lifetime risk of acquiring this malignancy. [1-3]. Peripheral nerve sheath tumours can be confirmed on US when the lesion is arising from a nerve, but clinical assessment is also vital [4, 5]. Additional investigations should be conducted if the lesion is painful, growing rapidly or there is distal neurological dysfunction. Further imaging is usually also required in patients with NF1. [6] Parameters in MRI that are associated with MPNST include irregular margins, perilesional edema, and necrosis [7].

In NF1, NF2, and schwannomatosis (SWN), emerging technical advances, particularly WB-MRI as well as DWI/ADC mapping, in conjunction with clinical and genetic data, can potentially provide insight into both disease severity as well as tumor behavior [8, 9]. To date, WB-MRI incorporating both anatomic sequences and DWI/ADC mapping is feasible and regularly utilized clinically for the assessment of patients with NF1, NF2, and SWN, enabling simultaneous detection and characterization of peripheral lesions. [10].

An acceptable accuracy for detecting malignant transformation of PNSTs in NF1 by the use of whole-body FDG-PET/CT has been demonstrated, combining SUV_{max} and TL_{mean} [11]. However, WB MRI offers the advantage to avoid high cumulative doses of ionizing radiation, which is essential since the screening mostly starts in pediatric or adolescent patients. Similar accuracy in diagnosing malignant PNTS have been reported for whole-body FDG-PET/CT and whole-body MR imaging [12]. PET/CT and MRI hare considered complementary roles in MPNST evaluation: In several studies, PET was more sensitive while the MRI offered higher specificity [7, 13]. In a recent meta-analysis in NF 1 patients, the pooled estimate of sensitivity of FDG PET/CT to detect MPNSTwas 0.99 vs. MRI with 0.85. The pooled specificities were 0.53 vs. 0.85 [13]. The use of WB PET/MR combines morphologic information by MRI with metabolic information by PET [14, 15]. WB PET/MR compared to PET/CT allowed detection of PET avid lesions with high accuracy, resulting in reduction of radiation exposure of almost 50%, [14] and therefore was considered a feasible alternative [14, 15].

2.3. Atypical lipomatous tumor (ALT)/ well-differentiated liposarcoma (WDLS)

- Adipocytic tumors with the following features in MR (or CT) are suspicious for ALT/WDLS : Size >11cm, deep location, septa >2mm, septal enhancement, nodular areas. Location in the lower extremity also increases the likelihood for ALT/WDLS.
- For adipocytic superficial and extremity ALT, if not primarily resected, ultrasound follow-up is recommended.
- Adipocytic tumors that are located in the retroperitoneal region or regions in which the tumor cannot be resected with a sufficient margin are termed WDLS. In case they are not resected, unenhanced MRI, or CT is preferred at yearly intervals or at the time when there are defined patient reported outcome measures (PROM) such as the presence of increased pain, size or tethering.

In the latest WHO classification of soft tissue tumors of 2020, with further incorporation of genetic alterations and immunohistochemical markers, new entities within the group of adipocytic tumors have been defined and others have been re-classified [16]. A detailed description would be beyond the scope of these guidelines. Regarding atypical lipomatous tumor (intermediate dignity, tumors located in the extremities and superficial sites) vs. well-differentiated liposarcoma (malignant, tumors in the retroperitoneum or mediastinum or regions in which the tumor cannot be resected with a sufficient margin), it is taken into account that the prognosis of adipocytic tumors of comparable histology differs depending on their location within the body [16]. A new separate entity termed atypical spindle cell/pleomorphic lipomatous tumour (benign, most common in hands and feet) has been defined[16].

For the differentiation of lipoma and ALT/WDLS, the following MR features seen can be helpful: Tumor location (lower extremity), deep or intramuscular location, size (> 11 cm), thick septa (> 2 mm), and enhancement of septa or nodular lesions [17-19] . Small pre-operative biopsies maybe misleading. If surgery is likely to lead to morbidity, radiological surveillance or watchful waiting can be considered [20].

References

1. *Miettinen MM, Antonescu CR, Fletcher CDM, et al. (2017) Histopathologic evaluation of atypical neurofibromatous tumors and their transformation into malignant peripheral nerve sheath tumor in patients with neurofibromatosis 1-a consensus overview. Hum Pathol 67:1-10*
2. *Tora MS, Xenos D, Texakalidis P and Boulis NM (2020) Treatment of neurofibromatosis 1-associated malignant peripheral nerve sheath tumors: a systematic review. Neurosurg Rev 43(4):1039-1046*
3. *Vasconcelos RAT, Coscarelli PG, Alvarenga RP and Acioly MA (2017) Malignant peripheral nerve sheath tumor with and without neurofibromatosis type 1. Arq Neuropsiquiatr 75(6):366-371*
4. *Noebauer-Huhmann IM, Vanhoenacker FM, Vilanova JC, et al. (2023) Soft tissue tumor imaging in adults: European Society of Musculoskeletal Radiology-Guidelines 2023-overview, and primary local imaging: how and where? Eur Radiol*
5. *Noebauer-Huhmann IM, Weber MA, Lalam RK, et al. (2015) Soft Tissue Tumors in Adults: ESSR-Approved Guidelines for Diagnostic Imaging. Semin Musculoskelet Radiol 19(5):e1*
6. *Reynolds DL, Jr., Jacobson JA, Inampudi P, Jamadar DA, Ebrahim FS and Hayes CW (2004) Sonographic characteristics of peripheral nerve sheath tumors. AJR Am J Roentgenol 182(3):741-4*
7. *Broski SM, Johnson GB, Howe BM, et al. (2016) Evaluation of (18)F-FDG PET and MRI in differentiating benign and malignant peripheral nerve sheath tumors. Skeletal Radiol 45(8):1097-105*
8. *Well L, Salamon J, Kaul MG, et al. (2019) Differentiation of peripheral nerve sheath tumors in patients with neurofibromatosis type 1 using diffusion-weighted magnetic resonance imaging. Neuro Oncol 21(4):508-516*
9. *Wilson MP, Katlariwala P, Low G, et al. (2021) Diagnostic Accuracy of MRI for the Detection of Malignant Peripheral Nerve Sheath Tumors: A Systematic Review and Meta-Analysis. AJR Am J Roentgenol 217(1):31-39*
10. *Ahlawat S, Blakeley JO, Langmead S, Belzberg AJ and Fayad LM (2020) Current status and recommendations for imaging in neurofibromatosis type 1, neurofibromatosis type 2, and schwannomatosis. Skeletal Radiol 49(2):199-219*
11. *Geitenbeek RTJ, Martin E, Graven LH, et al. (2022) Diagnostic value of (18)F-FDG PET-CT in detecting malignant peripheral nerve sheath tumors among adult and pediatric neurofibromatosis type 1 patients. J Neurooncol 156(3):559-567*
12. *Derlin T, Tornquist K, Munster S, et al. (2013) Comparative effectiveness of 18F-FDG PET/CT versus whole-body MRI for detection of malignant peripheral nerve sheath tumors in neurofibromatosis type 1. Clin Nucl Med 38(1):e19-25*
13. *Ko WS and Kim SJ (2023) Direct comparison of the diagnostic accuracy of 2-[(18)F]-fluoro-2-deoxy-d-glucose PET/CT and MRI for the differentiation of malignant peripheral nerve sheath tumour in neurofibromatosis type I: a meta-analysis. Clin Radiol*
14. *Raad RA, Lala S, Allen JC, et al. (2018) Comparison of hybrid 18F-fluorodeoxyglucose positron emission tomography/magnetic resonance imaging and positron emission tomography/computed tomography for evaluation of peripheral nerve sheath tumors in patients with neurofibromatosis type 1. World J Nucl Med 17(4):241-248*
15. *Reinert CP, Schuhmann MU, Bender B, et al. (2019) Comprehensive anatomical and functional imaging in patients with type I neurofibromatosis using simultaneous FDG-PET/MRI. Eur J Nucl Med Mol Imaging 46(3):776-787*
16. *WHO Classification of Tumours of Soft Tissue and Bone, 5th ed.* 2020 [cited 2023 14JUN]; Available from: https://publications.iarc.fr/Book-And-Report-Series/Who-Classification-Of-Tumours/Soft-Tissue-And-Bone-Tumours-2020.

17. *Asano Y, Miwa S, Yamamoto N, et al. (2022) A scoring system combining clinical, radiological, and histopathological examinations for differential diagnosis between lipoma and atypical lipomatous tumor/well-differentiated liposarcoma. Sci Rep 12(1):237*
18. *Nagano S, Yokouchi M, Setoguchi T, et al. (2015) Differentiation of lipoma and atypical lipomatous tumor by a scoring system: implication of increased vascularity on pathogenesis of liposarcoma. BMC Musculoskelet Disord 16:36*
19. *Wilson MP, Haidey J, Murad MH, Sept L and Low G (2023) Diagnostic accuracy of CT and MR features for detecting atypical lipomatous tumors and malignant liposarcomas: a systematic review and meta-analysis. Eur Radiol 33(12):8605-8616*
20. *Dangoor A, Seddon B, Gerrand C, Grimer R, Whelan J and Judson I (2016) UK guidelines for the management of soft tissue sarcomas. Clin Sarcoma Res 6:20*

Section 3. Pitfalls

3.1. Soft tissue masses simulating tumors

- Soft tissue masses simulating tumors are common and should be kept in mind when evaluating US and MRI.
- Examples for such masses are anatomical variants, muscle related, inflammatory, infectious, traumatic (nerve, muscle, reactive) skin lesions, metabolic lesions, vascular lesions.
- CT scan can be a problem-solving modality in benign entities and tumour mimickers.
- Pitfalls include myositis ossificans or osseous entities which cause high-grade soft tissue reaction, such as osteoid osteoma.

Soft tissue masses simulating tumors are common. The etiology is very broad, ranging from trauma, metabolic, reactive, anatomic variants, or other origins. The first imaging method should be US [1] and according to the possible non clear characterization, MRI should be further performed [2]. The list of masses simulating tumors could be classified as: a) Anatomical variants b) muscle related c) Inflammatory, infectious, d) traumatic (nerve, muscle, reactive) e) skin lesions, f) metabolic lesions, g) vascular lesions.
Sarcoma imaging compromises many challenging cases where the radiological findings can prevent unnecessary invasive procedures. In many of these cases a confident diagnosis can be achieved using CT. The decision to use CT to assess a soft tissue tumour is highly dependent upon the presented case scenario as well as the experience of the radiologist.
In soft tissue tumour imaging, challenging cases include myositis ossificans, or benign osseous entities which cause high-grade reactive changes of the adjacent soft tissue.
In cases of soft tissue tumour mimics such as myositis ossificans (focal myositis), CT (and US, CR) can provide crucial diagnostic information on the zonal distribution of the calcification and avoid unnecessary and potentially misleading biopsy [3, 4].
When close to joints or intra-articular, the nidus of an osteoid osteoma may be occult on MRI and the associated synovitis and joint distension on MRI may suggest an intra-articular proliferative disorder such inflammatory arthropathy or synovial chondromatosis.

3.2. Retroperitoneal liposarcoma

- Pitfalls in local soft tissue tumor staging include the assessment of the extension of well differentiated/dedifferentiated retroperitoneal liposarcoma: The well differentiated part of the tumor appears equivalent or similar compared to normal fatty tissue of the retroperitoneum both in CT and MRI. Comparison with the contralateral side can be helpful.

Well differentiated/dedifferentiated liposarcoma predominantly occurs in the retroperitoneum. Typically, the well differentiated areas of the tumor have a similar or identical appearance in CT and MRI, compared to the adjacent normal retroperitoneal fat [5]. Therefore, the evaluation of the tumor extension is often difficult. However, complete resection is crucial for the prognosis of the patient [6], as well-differentiated areas may undergo dedifferentiation. Comparison with the contralateral side can be helpful.

3.3. Consider potential multiplicity and syndromes

- Beware of satisfaction of search (SOS): Vascular anomalies, lipoma, lipoma of tendon sheath, desmoid, neurofibroma, myxoma, and inclusion body fibromatosis may be multiple.
- Mafucci's disease (hemangioma), Mazabraud (myxoma), Neurofibromatosis (schwannoma, neurofibroma), Gardner's syndrome (fibromatosis), Turner's syndrome (lymphangioma), Adenomatous poliposis (desmoid), Carney complex (myxoma) are syndromic associations with those of particular soft tissue lesions.

General approach in terms of preferred imaging modalities for soft tissue lesions can be followed regarding the location and the potential for malignant transformation (i.e. peripheral nerve sheath tumours in syndromic cases).

It is important to beware of satisfaction of search (SOS): Vascular anomalies [7], lipoma [8], lipoma of tendon sheath, desmoid [9], neurofibroma [10], myxoma, and inclusion body fibromatosis may be multiple. Maffucci's disease (hemangioma), Mazabraud (myxoma), Neurofibromatosis (schwannoma, neurofibroma), Gardner's syndrome (fibromatosis), Turner's syndrome (lymphangioma), Adenomatous polyposis (desmoid), Carney complex (myxoma) are syndromic associations with those of particular soft tissue lesions [10, 11].

References

1. *Aparisi Gomez MP, Errani C, Lalam R, et al. (2020) The Role of Ultrasound in the Diagnosis of Soft Tissue Tumors. Semin Musculoskelet Radiol 24(2):135-155*

2. *Perdikakis E, Tsifountoudis I, Kalaitzoglou I, Rountas C, Malliaropoulos N and Maffulli N (2017) Soft tissue pseudotumours: a pictorial review with emphasis on MRI. Muscles Ligaments Tendons J 7(2):353-375*
3. *Chai JW, Hong SH, Choi JY, et al. (2010) Radiologic diagnosis of osteoid osteoma: from simple to challenging findings. Radiographics 30(3):737-49*
4. *Lacout A, Jarraya M, Marcy PY, Thariat J and Carlier RY (2012) Myositis ossificans imaging: keys to successful diagnosis. Indian J Radiol Imaging 22(1):35-9*
5. *Murphey MD, Arcara LK and Fanburg-Smith J (2005) From the archives of the AFIP: imaging of musculoskeletal liposarcoma with radiologic-pathologic correlation. Radiographics 25(5):1371-95*
6. *Singer S, Antonescu CR, Riedel E and Brennan MF (2003) Histologic subtype and margin of resection predict pattern of recurrence and survival for retroperitoneal liposarcoma. Ann Surg 238(3):358-70; discussion 370-1*
7. ISSVA classification for vascular anomalies 2018. https://www.issva.org/UserFiles/file/ISSVA-Classification-2018.pdf.
8. *Gupta P, Potti TA, Wuertzer SD, Lenchik L and Pacholke DA (2016) Spectrum of Fat-containing Soft-Tissue Masses at MR Imaging: The Common, the Uncommon, the Characteristic, and the Sometimes Confusing. Radiographics 36(3):753-66*
9. *Desmoid Tumor Working G (2020) The management of desmoid tumours: A joint global consensus-based guideline approach for adult and paediatric patients. Eur J Cancer 127:96-107*
10. *Coffin CM, Davis JL and Borinstein SC (2014) Syndrome-associated soft tissue tumours. Histopathology 64(1):68-87*
11. *Perry HD and Cossari AJ (1986) Chronic lymphangiectasis in Turner's syndrome. Br J Ophthalmol 70(5):396-9*
